# Augmenting Mental Health Support for Patients Accessing Different Degrees of Formal Psychiatric Care through a Supportive Text Messaging Program: Protocol for a Randomized Controlled Trial

**DOI:** 10.3390/mps6010019

**Published:** 2023-02-13

**Authors:** Raquel da Luz Dias, Reham Shalaby, Belinda Agyapong, Gloria Obuobi-Donkor, Medard K. Adu, Ejemai Eboreime, Samuel Obeng Nkrumah, Sanjana Sridharan, Patryk Simon, Bryanne Taylor, Neal Henderson, Mathew D. White, Hugh Maguire, Gerald Gray, Faisal Rahman, Janah Fair, Nadine Wadden, Mutiat Sulyman, Olugbenga Williams, Oluseye Akinkunmi, Dorothy Edem, Pamela Arenella, Jason Morrison, Mahmoud Awara, Anand Natarajan, Abraham Nunes, Tomas Hajek, Claire O’Donavan, Rudolf Uher, JianLi Wang, Benjamin Rusak, Lori Wozney, Tara Sampalli, Doris Grant, Gail Tomblin Murphy, Jordan Warford, Samantha Hodder, Rachel Boe, Vincent Israel Opoku Agyapong

**Affiliations:** 1 Department of Psychiatry, Nova Scotia Health, Halifax, NS B3H 2E2, Canada; 2 Department of Psychiatry, Faculty of Medicine, Dalhousie University, Halifax, NS B3H 2E2, Canada; 3 Department of Psychiatry, Faculty of Medicine and Dentistry, University of Alberta, Edmonton, AB T6G 2B7, Canada; 4 Mental Health and Addictions Program, Nova Scotia Health, Halifax, NS B3S 1B8, Canada; 5 Department of Community Health and Epidemiology, Faculty of Medicine, Dalhousie University, Halifax, NS B3H 4R2, Canada; 6 Nova Scotia Health Innovation Hub, Halifax, NS B3S 0H6, Canada; 7 Mental Health and Addictions Program, IWK Health, Halifax, NS B3K 6R8, Canada; 8 School of Nursing, Cape Breton University, Cape Breton, NS B1M 1A2, Canada; 9 Addictions and Mental Health, Horizon Health Network, Fredericton, NB E3B 4R3, Canada

**Keywords:** mental health, cognitive–behavioural therapy, text messaging, e-health, health services, hospital admission, community mental health services

## Abstract

Patients feel more vulnerable when accessing community mental health programs for the first time or after being discharged from psychiatric inpatient units. Long wait times for follow-up appointments, shortage of mental health professionals, lack of service integration, and scarcity of tailored support can weaken their connection to the health care system. As a result, patients can present low adherence, dissatisfaction with treatment, and recurrent hospitalizations. Finding solutions to avoid unnecessary high-cost services and providing tailored and cost-effective mental health interventions may reduce the health system burden and augment patient support. We propose implementing an add-on, supportive text messaging service (Text4Support), developed using cognitive–behavioural therapy (CBT) principles to augment mental health support for patients attending to or being discharged from psychiatric care in Nova Scotia, Canada. This randomized controlled trial aims to investigate the effectiveness of Text4Support in improving mental health outcomes and overall mental well-being compared with usual care. We also will examine the intervention’s impact on health services utilization and patient satisfaction. The results from this study will provide evidence on stepped and technology-based mental health care, which will contribute to generating new knowledge about mental health innovations in various clinical contexts, which is not only helpful for the local context but to other jurisdictions in Canada and abroad that are seeking to improve their health care.

## 1. Introduction

Psychiatric patients may experience increased vulnerability during specific periods of their journey, especially when they first enrol in a mental health program or after being discharged from an inpatient unit [1,2]. Long wait times for follow-up appointments, shortage of mental health professionals, lack of service integration, and scarcity of tailored support can weaken the connection with the health care system for both newly admitted outpatients and those released from inpatient facilities [3]. As a result, patients can present dissatisfaction and low adherence to treatment, which may lead to undesirable outcomes such as mental health deterioration, increased readmissions, recurrent emergency department (ED) visits, and extended lengths of stay (LOS) during hospitalization [4,5]. Alleviating potential health distress and strengthening the patient’s connection with the health care service and provider can close the treatment gap experienced by many patients accessing or being discharged from formal mental health care services [6]. Moreover, delivering appropriate support and engaging patients with their treatment, especially when they feel vulnerable, are critical strategies for the effectiveness of mental health care and may positively impact health services utilization, preventing unnecessary high-cost health service use [6].

However, overcoming the gap in scaling up patient support should be not only evidence-based but cost-effective. One solution that meets these requisites is text message interventions. Short message service (SMS), commonly referred to as “text messaging,” is a service for sending short messages of up to 160 characters to mobile devices [7]. Even though this technology was developed more than 30 years ago, and even though it may be considered outdated in comparison to more cutting-edge technologies (e.g., mobile apps, augmented reality, etc.), text messages are now a part of the vast majority of people’s daily lives, becoming the most common and affordable communication function used on mobile phones, which is one of the reasons why they are still used to deliver virtual care in a significant portion of e-health services, including mental health and addiction programs (MHAPs) [8].

Text messaging technology has been proposed as a means to augment and extend the reach of mental health support [9,10]. Brief texting can be used as supportive text messages, social support engagement, care team contact capabilities, data feedback, psychoeducation, adherence-based psychotherapy, remote care delivery, medication adherance, and contingency management [11].

Cognitive–behavioural therapy (CBT), the standard of care for numerous mental health conditions, can also be delivered through text messaging programs [12]. Text4Mood and Text4Hope are two examples of such initiatives that have been successfully implemented in Alberta, Canada. Both programs have demonstrated that this innovation mitigates geographical limitations to access, offering a convenient and accessible means to provide mental health support for patients seeking psychological services as well as at the population level. Text messages were also to be found cost-effective since they do not require technical expertise, are free for end users, and only cost providers cents for each delivered message [13,14,15].

Two randomized controlled trials (RCTs) have also examined the efficacy of these interventions. The first one aimed to investigate the efficacy of supportive text messages in improving treatment outcomes in patients with major depressive disorder (MDD) [16]. For three months, the patients in the intervention group (*n* = 35) received twice-daily supportive text messages, whereas those in the control group (*n* = 38) received a single text message every two weeks, thanking them for taking part in the study. The Beck’s Depression Inventory-II (BDI-II) scores corrected for baseline showed a significant difference between the intervention and control groups at three months: 20.8 (SD = 11.7) vs. 24.9 (SD = 11.5), F (1, 60) = 4.83, *p* = 0.03, and *p*2 = 0.07, with an effect size (Cohen’s d) of 0.67. Similar differences (also adjusted for baseline scores) were observed between the intervention and control groups for the three-month mean self-rated overall health status scores (EQ-5D-5 and EQ-5D VAS): 65.7 (SD = 15.3) vs. 57.4 (SD = 22.9), F (1, 60) = 4.16, *p* = 0.05, and *p*2 = 0.065, with an effect size (Cohen’s d) of 0.51. These results indicate the potential value of supportive text messages as a psychological treatment for depression, particularly in underserved populations. The intervention was also found to reduce the psychological treatment gap, with 82% of respondents to a user survey reporting that the text messages made them more hopeful about dealing with issues in their lives. Additionally, 77% felt in charge of managing their depression and anxiety symptoms owing to the SMS messages, and 75% felt connected to a support network. The majority of the responders (83%) said Text4Mood enhanced their general mental well-being, supported their follow-up care (52.5%), and increased their sense of belonging (75.2%), all of which may have an influence on the demand for more intensive therapies [16].

Another RCT evaluated the effectiveness of Text4Hope, a CBT-based text message intervention deployed during the COVID-19 pandemic targeting the general population’s mental well-being [17]. The results from this trial indicated that receiving text messages for a brief period of time (six weeks) was effective in reducing stress, anxiety, and depression symptoms, as well as suicidal ideation in subscribers. The sum of the mean scores on validated scales such as the Perceived Stress Scale (PSS), the Generalized Anxiety Disorder-7 (GAD-7) scale, and the Patient Health Questionnaire-9 (PHQ-9) were compared. Subscribers who had received the messages for six weeks scored 20.9% lower than those who had recently subscribed to the program [17]. With >54,000 subscribers from March 2020 to July 2021, this emergency intervention proved to be scalable and significantly mitigated the need for readmission, a risk for COVID-19 infection, by reducing mental health distress by over 20% during the pandemic [18].

Text4Support is another text messaging program that offers mental health and addiction patients daily supportive text messages tailored to their diagnosis or most significant mental health concerns. The text messages have been developed by psychiatrists, psychologists, mental health therapists, and service users using CBT principles by focusing on the cognitive triangle of thoughts, feelings, and behaviours. The Text4Support suite of programs includes banks of messages, each of which is targeted to a specific concern: general well-being, depression, bipolar disorder, anxiety, psychosis, substance use disorder, personality disorders, and trauma-related disorders. A retrospective analysis of survey responses from individuals seeking access to mental health and addiction services (*n* = 296) enrolled in the Text4Support program for six months revealed that the text messaging intervention is a viable tool to complement existing programs and increase patient-system contact [19]. Another study used a combination of methods to assess user satisfaction with the Text4Support program [20]. Qualitative data collected from individual interviews and quantitative information extracted from web-based survey responses of the patients (*n* = 181) discharged from acute psychiatric care revealed that Text4Support was well received, with a high satisfaction rate for either message feedback or perceived impact. Furthermore, subscribers were pleased with the frequency of the messages, which were delivered once daily for six months. As a result of participant suggestions, the most recent version of the program includes separate text message banks targeting specific mental health disorders.

Although many studies [21,22,23,24,25,26] have shown that text messaging programs can improve the mental health of a varied population, when it comes to the specific diagnoses of psychiatric conditions and different levels of care complexity, the only data available come from pilot studies limited to patients diagnosed with depression and enrolled in outpatient clinics. This proposed trial primarily aims to close this evidence gap by providing fresh data on the efficacy of a CBT-based supportive text messaging program (Text4Support), compared with usual care, in improving transdiagnostic mental health outcomes among patients accessing different degrees of formal mental health care. Secondarily, we want to look at how the intervention affects the use of health services (crisis calls, ED visits, admissions and readmissions, and LOS following inpatient hospitalization) and patient satisfaction with mental health services (overall satisfaction, retention and withdrawal rates, and patient engagement). Lastly, we will explore Text4Support implementation outcomes (reach of the intervention, acceptability, appropriateness, fidelity, and cost-effectiveness). This research protocol will be helpful for any mental health services looking to enhance patient care and health services utilization while also extending support for those who need it. The findings of this study are expected to advance our understanding of mental health innovations in a variety of clinical contexts.

## 2. Materials and Methods

### 2.1. Study Design

This randomized controlled trial (RCT) is a multicenter, longitudinal, prospective, parallel, two-arm, rater-blinded methodology, with an embedded one-phase patient satisfaction qualitative sub-study and an implementation evaluation component. Hence, the study will have two groups (i.e., intervention group (IG) and control group (CG)), to which study participants will be randomized. The randomization process will employ permuted blocks, to ensure balance (1:1) between groups. To ensure allocation concealment, simple random allocation will be performed using a random number generator. The primary outcome measure for this study is transdiagnostic, with recruitment occurring in all mental health clinics and programs across the province, so stratified sampling will not be employed. Research team members working on the randomization and allocation processes will not be involved in the baseline and follow-up assessments. Data analysts will be blinded to treatment group allocation. Study subjects will, however, not be blinded since it is impossible to conceal which experimental condition they will be assigned to.

The qualitative sub-study on patient satisfaction will utilize qualitative descriptive methodology and thematic analysis [27]. We will select a purposive sample of the research participants to attend focus group sessions to uncover patterns or themes in the patient experience and satisfaction with the interventions. Finally, guided by the RE-AIM framework [28], we will evaluate the success of Text4Support implementation by looking at the intervention’s reach, effectiveness, willingness to adopt it, fidelity, and sustainability post-trial.

**Figure 1:**
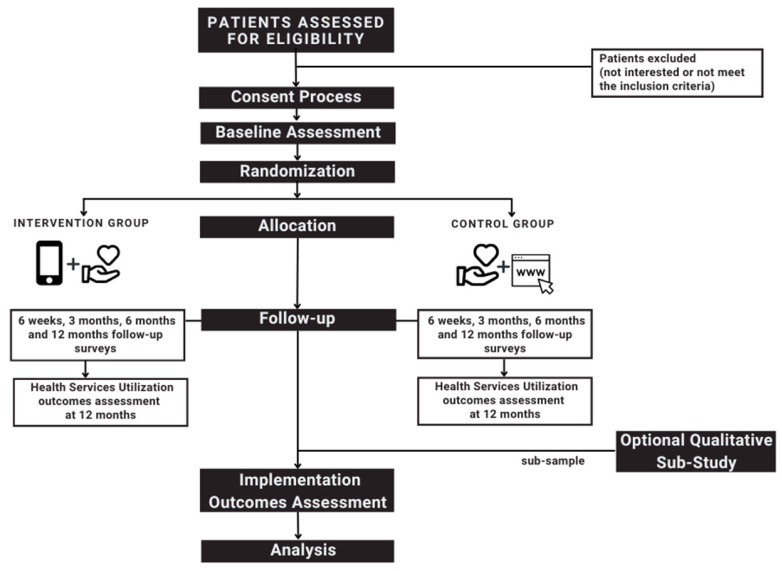
Study flow diagram.

This protocol has been registered at ClinicalTrials.gov (NCT05411302) and approved by the Research Ethics Board for Nova Scotia Health (REB File#1028174). The trial will be carried out over three years, with 24 months dedicated to recruitment. The intervention will be delivered for 6 months, followed by an observation period of 12 months for each participant. The study flow diagram is represented in Figure 1.

### 2.2. Settings and Study Participants

The study will be conducted at mental health and addiction (MHA) outpatient and inpatient services, including community mental health and addiction programs (CMHAPs), specialty programs (SPs), addiction programs (APs), emergency departments (EDs), and psychiatric inpatient units (PIUs), across the province of Nova Scotia, Canada. The recently launched Day Hospital and the Transcultural Mental Health Program, as well as the Rapid Access and Stabilization Program that is expected to be initiated, will be part of the study, totalling 46 study sites. New patients (i.e., first-time users) of MHA outpatient programs and patients being discharged from MHA inpatient facilities (i.e., at most one week prior to their expected discharge date) will be eligible for the study. Additionally, they must be 18 years old or older, have a phone capable of receiving text messages (no data plan is required), be able to read English, and provide written consent. Patients will be disqualified if they are unable to read text messages from a mobile device or are unwilling or unable to grant permission to participate in the trial.

### 2.3. Sample Size Considerations

With a hypothesized difference of 20% in change scores (from baseline to the four-time points) for each of the primary outcome variables of interest (e.g., mean change score of 10 vs. 8), a population variance of 5.0 for the mean change score, a 2-sided significance level (α = 0.05), a power of 90% and an acceptable difference between the sample mean and population mean score for each scale of zero (μ − μ0 = 0), an estimated sample size of 131 participants in each arm of the study would be needed (based on the 6Sigma online script) to detect the projected mean differences in mean change scores between the intervention and control groups [29,30]. Thus, the sample size for the RCT would be 262. Given that the maximum survey completion rate for the online surveys delivered via text message is approximately 20%, in order to achieve 262 completed survey responses at each time point, we plan to enrol at least 1500 participants in the RCT. This recruitment rate is achievable since a related study in Alberta [31] currently has over 500 patients recruited within six months. The sample size for the qualitative component of the study cannot be predetermined. Data saturation is reached across data analysis, ending when no new themes and subthemes emerge. However, based on what the literature suggests [32,33], we anticipate that 25–30 participants may be sufficient for this particular mixed-method study.

### 2.4. Study Procedures

#### 2.4.1. Consent Process

Patients from all study sites will be initially provided with information about the study by someone from their circle of care. A research team member will facilitate the study presentation, consent process, and study enrolment on the day of the patient’s enrolment in the MHA outpatient program or within a week before their expected discharge date from the inpatient units. A REDCap-based electronic consent form (e-Consent) will be used to obtain consent. The person obtaining consent will gain access to the information leaflet about the study and the e-Consent by pointing a mobile device camera (tablet supplied by the study or patient’s mobile phone) to a QR Code, which will open the form link. During the informed consent process, patients will receive detailed information about the study’s goal and procedures, risks and benefits, and participation alternatives. They can choose whether or not to participate in the RCT and/or the qualitative sub-study by providing consent or not. Participants will be advised about the voluntary nature of the study and that they may withdraw from the study at any time they want without giving a reason. A digital copy of the information leaflet will be sent to the participant’s email address at the completion of the consent procedure.

#### 2.4.2. Baseline Assessment

After providing consent, all subjects will be asked to complete an online baseline survey. The baseline survey includes sociodemographic information and self-completed validated scales designed to assess the patient’s mental health outcomes. During the baseline assessment, participants will be given the option of selecting the message bank from which they want to receive text messages based on their needs (i.e., depression, anxiety, bipolar disorder, etc.). In addition to that, one clinician-rated scale, which evaluates suicidal risk, will be completed by a trained research team member in person or over the phone. The average time for completing the baseline survey is 10 min.

#### 2.4.3. Randomization and Allocation

Patients who provided consent will be enrolled in the study after completing the online baseline survey and entering a mobile phone number in the space provided. Participants will be randomly assigned to the IG or CG according to the methodology previously described.

#### 2.4.4. Follow-Up Assessment

Participants in both study arms (intervention and control) will receive a link to the follow-up self-completed online surveys through SMS and be asked to complete them at 6 weeks and 3, 6, and 12 months after enrolment in the study. The same clinician-rated scales completed at enrolment will be completed over the phone by a trained research team member. A monitoring compliance strategy will be put into practice (a reminder text message after two days and then phone calls from the research team) in order to mitigate the loss of follow-up and improve patient compliance.

#### 2.4.5. Optional Qualitative Sub-Study

A sub-sample of the study population will be invited to participate in individual interviews and focus groups. As this qualitative study does not aim to generalize findings but rather to provide depth, a purposive sampling approach will be employed to select the participants. The mental health program the participant is subscribed to, which is an indication of the primary presenting problems or diagnosis, will be considered in the selection of participants for the qualitative sub-study. In addition, sex- and gender-based representation, age groups, and marginalized groups will be considered in the sample, ensuring that a diverse group is represented in this sample. The method used to draw a heterogeneous sample will be by extracting from the database the unique study IDs and the respective mental health program, primary presenting problem/diagnosis, age, sex, gender, and ethnicity information. An Excel spreadsheet will be created for each variable, and a random sampling method will be run for each one. After drawing the sub-sample, potential participants will be invited to the sub-study via a text message. If the estimated number of participants cannot be reached after the invitations, another round of sampling and invitations will be carried out. The inclusion criteria to take part in this qualitative sub-study are having previously provided consent and being available to attend a 2 h, audio-recorded, individual interview and focus group session. The participants of the optional qualitative sub-study will be reminded of their consent, possible risks (feeling uncomfortable during the individual interviews and/or focus group while discussing their experiences with the mental health services, and risk of participant identification), and possible benefits (e.g., results may contribute to improvement in mental health services). A trained research team member will conduct the meetings in person or via Zoom Healthcare. The focus group will have a maximum of 10 participants and will last less than 1.5 h. Participants in the optional qualitative sub-study will be compensated with a gift card for their time spent in the research activities.

### 2.5. Proposed Interventions

**Figure 2:**
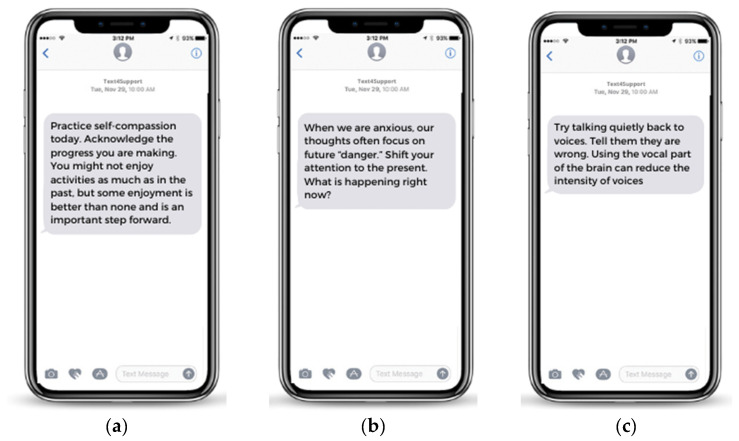
(**a**–**c**): Text4Support general well-being message; Text4Support anxiety message; Text4Support psychosis message.

The intervention to be tested in this trial is the Text4Support program, which will be compared with a control condition. A day after enrolment, the participants randomized to the IG (Text4Support) will start receiving daily unidirectional (no-reply), CBT-based, diagnostic-specific supportive text messages for 6 months, as an add-on program to their usual care, with a subsequent observation period of 6 months, totaling 12 months of follow-up. Text4Support is powered by ResilienceNHope [34], an online application offered by the Global Psychological eHealth Foundation [35]. In action, Text4Support provides general content indicated regardless of the symptomatology presented, including messages of self-care, social support, hope, affirmation, and recovery (Figure 2a), as well as diagnostic-specific content focused on managing symptoms related to the diagnosis, such as the examples shown in Figure 2b,c. It is possible to observe the principles of the CBT cognitive triangle (thoughts, feelings, and actions) in the text messages shown, which is a key component of the program’s therapeutic effect. The text messages always encourage the recipients to consider their thoughts and feelings, and they suggest actions to take. The text message bank includes 180 messages per category (i.e., stress, mood disorders, anxiety disorders, depression, schizophrenia and other psychotic disorders, substance use disorders, adjustment disorders, and personality disorders). The messages were originally developed in Canadian English, and while the online application allows for translation into other languages such as French, Punjabi, Arabic, Ukrainian, Russian, and simplified Chinese, the proposed program will only be delivered in English. The program targets individuals who are receiving any type of assessment and/or treatment from mental health and addiction services. The subscribers can choose the bank of messages they want to receive based on their primary mental health concerns. The possibility of covering a wide range of mental health conditions, from the most to the least frequent and from mild to critical disorders, means that it can be used to augment mental health support for patients receiving various levels of mental health care in accordance with the project’s goals.

The control condition comprises the standard care offered by each program/unit participating in this study (i.e., follow-up appointments or offering of community clinic/program treatment) and a single text message with the link to the e-mental health resources available on the NS Health MHAP website [36], following 12 months of the observational period. Currently, the NS Health MHAP website offers 22 freely accessible, evidence-based e-mental health tools covering a wide range of psychiatric conditions (i.e., substance abuse disorders, gambling, depression, anxiety, grief, bipolar disorders, eating disorders, obsessive–compulsive disorder, post-traumatic stress disorder, schizophrenia, and psychosis, among others), group ages (children, youth, adult, and seniors) and group identities (Black/African Nova Scotians, 2SLGBTQIA+, Canadian Forces, RCMP, caregivers and families, First Nations/Indigenous people, and first responders).

### 2.6. Outcome Measures

The intervention effectiveness will be determined by evaluating the differences between the intervention and control group from baseline and the subsequent time points (6 weeks and 3, 6, and 12 months) for patient clinical outcomes and the differences between groups for health service utilization at 12 months, and the implementation success will be measured through quantitative and qualitative patient satisfaction data and implementation indicators, as follows:

- Clinical outcomes: The primary clinical outcome will be the differences in mean change in patient recovery and quality of life, assessed through the Recovery Assessment Scale (RAS) [37] and the World Health Organization Five Well-Being Index (WHO-5) [38]. Secondary clinical outcomes will include the differences between the intervention and control groups in prevalence changes for moderate-to-high depression (likely major depressive disorder), moderate-to-high anxiety (likely generalized anxiety disorder), and low resilience from the baseline to these time points, assessed through the Patient Health Questionnaire-9 (PHQ-9) [39], Generalized Anxiety Disorder-7 scale [40], and the Brief Resilience Scale (BRS) [41]. The mean score differences between groups from the baseline to determine time points will also be assessed using the Adverse Childhood questionnaire (ACE questionnaire) [42], and the Brief Substance Use Craving Scale (BSCS) [43], which will be used to assess childhood trauma and substance craving, as well as the clinician-rated Columbia-Suicide Severity Rating Scale (C-SSRS) [44], which will be used to assess suicide risk.
- Health service utilization outcomes: Health service utilization data will be extracted by the registration, reporting, and analytics, mental health and addiction team from the multiple health information systems utilized across NS Health and provided to the research team through reports. The differences in health service utilization data (mobile crisis visits, number of ED presentations, number of admissions in the last 12 months, length of stay, and the number of hospital readmissions) will be evaluated for participants in both groups. The differences between groups will be evaluated at 12 months.
- Patient experience and satisfaction outcomes: Patient satisfaction and patient experience with the e-mental health interventions (Text4Support and other e-mental health programs offered freely through NSH Authority) will be evaluated using both quantitative and qualitative data. Overall satisfaction will be assessed at each of the four time points (6 weeks and 3, 6, and 12 months) using a self-designed scale. Self-reported retention and withdrawal will both be assessed through the satisfaction survey, which addresses questions about the frequency of reading messages, the frequency of returning to messages stored in their phones, and actions taken by the participants after reading the text messages or freely available e-mental health resources (i.e., text “STOP”, clicking on links, searching for more information) up to 12 months after enrolment. Patient experience and satisfaction qualitative data will be collected through individual interviews and focus groups.
- Implementation Outcomes: Implementation outcomes are indicators of implementation efforts and are distinct from service or clinical outcomes [45]. For the intervention arm, we will include the reach of the interventions, acceptability, appropriateness, fidelity, and cost-effectiveness, evaluated at each of the four time points [28]. These implementation indicators will not be collected from the e-mental health or website visits in the control arm because they are not tied to the patient’s study ID and cannot be linked to the other measures.

Quantitative clinical and health services utilization outcome measures and time points are detailed in Table 1.

### 2.7. Data Analysis

Quantitative data will be analyzed in concordance with the CONSORT guidelines [46]. The baseline demographic and clinical characteristics of the two groups will be analyzed using Chi-squared, Fisher’s Exact, and Student’s *t*-tests. The primary (clinical mental health outcomes) and secondary outcome (health services utilization) measures will be analyzed using descriptive and inferential statistical analysis, and Bonferroni correction will be used when necessary. The scores at each time point on standardized rating scales for clinical variables (PHQ-9, GAD-7, WHO Five Well-Being Index, BRS, RAS, ACE, C-SSRS, and BSCS) will each be compared between the intervention and control groups using an analysis of covariance (ANCOVA) with the treatment condition as the independent variable, the baseline scores on clinical variables as the covariate, and the scores at each time point on standardized rating scales as the dependent variable. In each case, checks would be conducted to ensure the reliable measurement of the covariate and no violation of the statistical assumptions of normality, linearity, homogeneity of variance, and homogeneity of regression slopes. Multiple imputations will be our baseline approach to addressing missing data, although analysis of missingness patterns must be performed to inform our specific assumptions, implementation, and interpretation during missing data analysis. Sensitivity analyses will also be conducted with respect to the imputation approach, including a comparison with the last observation carried forward, mean/median imputation, best case, worst case, and complete case analyses. The reasons for discrepancies between approaches will be analyzed and discussed in the study manuscript. We will also compare the prevalence scores for the clinical variables of interest using Chi-square/Fisher’s exact tests. To explore the impact of select demographic and clinical variables on the clinical variables of interest, we will perform a two-way between-groups = analysis of variance. Characteristics of individuals/clusters will be summarized by exposure status to allow the consideration of selection biases and lack of balance [47]. In terms of the number of multiply imputed datasets to be analyzed, we will begin with 100 datasets. A sensitivity analysis will subsequently increase this number to ascertain the stability of model estimates in relation to the number of multiply imputed datasets.

Patient satisfaction quantitative data will be analyzed by using one-way analysis of variance (ANOVA) tests to compare satisfaction between the two study groups. Likert scale satisfaction responses to various aspects of Text4Support and freely accessible Health-Authority-approved low-intensity e-mental health services will be summarized as frequency counts of response categories and percentages. We will compare the differences in satisfaction between the groups by using the Chi-square/Fisher exact test with two-tailed criteria. A Bonferroni-corrected, two-tailed criterion (α = 0.05/*n* (*n* = number of comparisons) will be used to determine statistical differences.

Qualitative data analysis will be aligned with qualitative descriptive methods. The qualitative data analysis will be guided using the six-phase thematic analysis framework [26] and conducted by two distinct members of the research team. They will perform an independent analysis and then compare their findings. Data disagreements will be resolved by discussion and convergence, if possible. The remaining disagreements will be resolved by a third researcher and/or documented for discussion in the study report.

The first step of qualitative analysis is to transcribe verbatim all audio records from the semi-structured individual interviews and focus groups and enter them in NVIVO 12 [48] software for data organization and preparation. This step is crucial to becoming familiar with the data, and reading and re-reading the transcripts. At this stage, we will also perform data validation for quality assurance purposes. A data validator, who is a different person from the transcriber, will compare the verbatim transcriptions with the recorded interviews for verbal and non-verbal errors (commissions and omissions). Errors in transcription will be corrected during validation. The subsequent phases of thematic analysis (coding, searching for themes, themes review, themes definitions, and writing) will be accomplished [27]. The sample size for the qualitative sub-study will depend on data saturation [32,33].

The analysis of the implementation outcomes will be guided by the RE-AIM framework (reach of the interventions, acceptability, appropriateness, fidelity, and cost-effectiveness) [28]. We want to look into the context, method, and consequences of deploying Text4Support and compare them. The adoption of the Text4Support intervention will be evaluated using a combination of quantitative and qualitative data, as well as subsequent analysis (as mentioned in the preceding sections).

## 3. Expected Results

The results of this study are expected within three years after the project initiation. Based on previous research [13,14,15,16,17,18,19,20], we anticipate that Text4support in Nova Scotia will achieve similar mental health outcomes, with a 20% greater improvement in patients’ recovery and quality of life and a 20% greater reduction in depressive and anxiety symptoms, when assessed by the mean change in validated scale scores at six weeks and three, six, and twelve months from the baseline compared with control interventions. The mean differences in scores obtained from other validated scales that will assess resilience, childhood trauma, substance craving, the presence and severity of psychotic disorders, suicide risk, and manic symptoms are expected to be alike. We also anticipate a 10–25% reduction in high-cost health service use (psychiatric readmissions and ED visits) among Text4Support participants compared with the usual care group. An unpublished pilot trial in Alberta (*n* = 180) found that the patients discharged from inpatient psychiatric units who received Text4Support messages for 6 months made 10% fewer ED visits in the 6 months following discharge than a control population [6]. We anticipate that Text4Support participants will also use fewer high-cost health services in Nova Scotia. In terms of the retention and patient engagement rates, we confidently predict that IG participants will achieve higher figures (at least 80%) than the CG participants’ self-reported retention rate and engagement on e-mental health resources. This optimistic estimation is based on evidence of “push” text message technologies, in which intervention messages are delivered without the individual’s intervention and have a 98% open rate [49]. Additionally, previous research has shown that after six weeks of intervention, the retention rate in similar text messaging programs (Text4Hope) was around 90% [17,18], indicating that subscribers were still opening and reading the messages.

## 4. Risks and Limitations

As with every research study, there is a risk of data identification. To prevent this from happening, all the collected data will be labelled with a unique study ID number. Only the aggregated and de-identified data will be used in publications. Regarding the intervention, the risk to participants is not supported by evidence from our predecessor programs, such as Text4Mood and Text4Hope, but even so, client safety and monitoring protocols will be implemented throughout the trial. The research team will monitor patients through the data collected and notify patients and healthcare providers if the results indicate a potential risk to the patient (e.g., if a patient presents an assessment score compatible with suicidal ideation). In this case, participants will be directed to call a crisis line or go to their local ED if an emergency occurs.

## 5. Conclusions

This study will demonstrate an innovative approach to augmenting mental health support to individuals accessing different degrees of formal psychiatric care in Nova Scotia. The success of Text4Support in the province will not only provide evidence relevant to new stepped care and technology-based approaches to mental health service delivery but will also contribute to the knowledge of how to implement mental health innovations in different clinical contexts, contributing to close the mental health support worldwide.

## Figures and Tables

**Table 1:** Text4Support RCT outcomes measures, instruments and data collection time points.

Outcome Measures	Instrument	Description	Data Collection Time Points				
			Baseline	6 w	3 m	6 m	12 m
***Clinical outcomes (measures of impact)***							
**Patient’s recovery**	Recovery Assessment Scale (RAS) [37] *	RAS is a 20-item measure developed based on a process model of recovery, which evaluates various aspects of recovery with a special focus on hope and self-determination.	X	X	X	X	X
**Quality of Life**	WHO-5 Well-Being Index [38] *	WHO-5-Well-Being Index is a short 5-item generic global rating scale measuring subjective well-being. The scale was derived from the WHO-10, which in turn was derived from a 28-item rating scale. The scale has adequate validity both as a screening tool for depression and as an outcome measure in clinical trials.	X	X	X	X	X
**Likely depression**	PHQ-9 [39] *	The PHQ-9 is a 9-item validated instrument used to diagnose and measure the severity of depression in general medical and mental health settings. The scale has good convergent validity and adequate internal consistency.	X	X	X	X	X
**Likely anxiety**	GAD-7 [40] *	GAD-7 is a validated 7-item questionnaire used to assess the self-reported levels of anxiety in respondents in the two weeks prior to assessment.	X	X	X	X	X
**Patient’s Resilience**	BRS [41] *	The BRS assesses the perceived ability to bounce back or recover from stress. The possible score range on the BRS is from 1 (low resilience) to 5 (high resilience)	X	X	X	X	X
**Patient’s trauma-related stress**	ACE questionnaire [42] *	The ACE Questionnaire is a 10-item measure used to measure childhood trauma. The questionnaire assesses 10 types of childhood trauma measured in the ACE Study.	X				
**Patient’s tendency and desire for each addictive substance**	BSCS [43] *	BSCS is a self-reporting scale to assess the intensity, frequency, and length of time spent craving in the past 24 h, using a five-point Likert scale ranging from 0 to 4 with a mean score of 0 indicating no cravings in the past 24 h and a mean score of 4 indicating a high tendency for drug craving.	X	X	X	X	X
**Patient’s suicidal risk**	Columbia Rating Scale [44] **	C-SSRS is a self-reporting suicidal ideation and behaviour rating scale to evaluate suicide risk.	X	X	X	X	X
***Health Services Utilization Outcomes (measures of impact)***							
**Crisis calls**	Administrative data	The number and proportion of patients calling the Crisis helpline.	-	-	-	-	X
**Hospital Admissions**	Administrative data	The number and proportion of MHA admissions into medical units, specialty, forensic of withdrawal management units in the last 12 months	-	-	-	-	X
**Length of Stay**	Administrative data	Length of stay for each MHA-related hospital admission.	-	-	-	-	X
**Hospital readmissions**	Administrative data	The number and proportion of patients readmitted into MHA acute care units within 30-days after discharge.	-	-	-	-	X
**Emergency Department (ED) presentations**	Administrative data	The number and proportion of MHA-specific ED presentations.					
***Implementation outcomes (measures of process)***							
**Reach**	Administrative data	The proportion of the target population who receive daily supportive text messages across Nova Scotia.	-	X	X	X	X
**Acceptability**	Self-designed instrument	Evaluates clients’ satisfaction and experiences with supportive text messaging programs.	-	X	X	X	X
**Appropriateness**	Sociocultural, gender and age sensitivity.	Qualitative in-depth interviews	-	X	X	X	X
**Incremental cost-utility**	Administrative data	The incremental cost-effectiveness ratio (ICER) is the ratio between the difference in costs and the difference in benefits of the intervention	-	X	X	X	X
**Overall satisfaction, retention, withdrawal, and engagement rate**	Self-designed Online survey	Evaluates clients’ satisfaction and experiences with supportive text messaging programs.		X	X	X	X

* Online, patient-completed scales. ** Clinician-rated scale.

## Data Availability

Data is available upon reasonable request to the submitting author.
